# Assessment of the Impact of Potential Tetracycline Exposure on the Phenotype of *Aedes aegypti* OX513A: Implications for Field Use

**DOI:** 10.1371/journal.pntd.0003999

**Published:** 2015-08-13

**Authors:** Zoe Curtis, Kelly Matzen, Marco Neira Oviedo, Derric Nimmo, Pamela Gray, Peter Winskill, Marco A. F. Locatelli, Wilson F. Jardim, Simon Warner, Luke Alphey, Camilla Beech

**Affiliations:** 1 Oxitec Ltd, Milton Park, Abingdon, Oxfordshire, United Kingdom; 2 Imperial College London, South Kensington, London, United Kingdom; 3 Chemistry Institute, Campinas University (UNICAMP), Campinas, São Paulo, Brazil; 4 Redox Consultoria e Remediação Ambiental, Itu, Sao Palo, Brazil; 5 Department of Zoology, Oxford University, Oxford, United Kingdom; University of Perugia, ITALY

## Abstract

**Background:**

*Aedes aegypti* is the primary vector of dengue fever, a viral disease which has an estimated incidence of 390 million infections annually. Conventional vector control methods have been unable to curb the transmission of the disease. We have previously reported a novel method of vector control using a tetracycline repressible self-limiting strain of *Ae*. *aegypti* OX513A which has achieved >90% suppression of wild populations.

**Methodology/Principal Findings:**

We investigated the impact of tetracycline and its analogues on the phenotype of OX513A from the perspective of possible routes and levels of environmental exposure. We determined the minimum concentration of tetracycline and its analogues that will allow an increased survivorship and found these to be greater than the maximum concentration of tetracyclines found in known *Ae*. *aegypti* breeding sites and their surrounding areas. Furthermore, we determined that OX513A parents fed tetracycline are unable to pre-load their progeny with sufficient antidote to increase their survivorship. Finally, we studied the changes in concentration of tetracycline in the mass production rearing water of OX513A and the developing insect.

**Conclusion/Significance:**

Together, these studies demonstrate that potential routes of exposure of OX513A individuals to tetracycline and its analogues in the environment are not expected to increase the survivorship of OX513A.

## Author Summary

Dengue fever is spread by the mosquito *Aedes aegypti* and the most effective method to limit the spread of dengue is to reduce the mosquito population. We have previously reported a transgenic strain of *Ae*. *aegypti* which results in >90% population suppression: males, which do not transmit disease, are released into the field carrying a self-limiting gene to mate with wild females, passing on the self-limiting gene which causes >95% progeny to die before becoming vectors of disease. To be able to breed this mosquito in the laboratory an antidote, tetracycline, is used to suppress the effects of the transgene. Given that tetracyclines are commonly used in human and veterinary medicine, it is essential to consider whether sufficient tetracycline could be in the environment to prevent the effective use of this control method by allowing the female’s progeny (from a mating between a released OX513A male and a wild female) to survive. Here we have shown that the concentrations of tetracycline to which the mosquitoes will be exposed in the environment, both in breeding sites and in a blood-meal host are not high enough to influence the effectiveness of this control method.

## Introduction

Dengue fever is a viral infection, primarily transmitted by the mosquito *Aedes aegypti*, with an estimated incidence of 390 million infections annually [1]. There are currently no licensed vaccines [2, 3] so control of the disease is focused on suppression of the vector. Commonly employed vector control techniques such as larval site removal, use of larvicides and insecticide fogging have been unable to provide satisfactory control of *Ae*. *aegypti* and dengue transmission.

Previously we have reported a novel method of vector control based on the sterile insect technique (SIT) [4–6]. In this approach, males homozygous for a tetracycline repressible transgene, which confers the self-limiting trait to progeny are released into the environment where they search for and mate with wild females. Offspring of these males inherit a single copy of the transgene and, lacking adequate exposure to the tetracycline repressor, >95% die before becoming adults and potential vectors of blood borne diseases. Using the OX513A strain of *Ae*. *aegypti*, this method resulted in the suppression of the wild *Ae*. *aegypti* population in East End, Grand Cayman [5] and in Itaberaba, Brazil.

**Fig 1:**
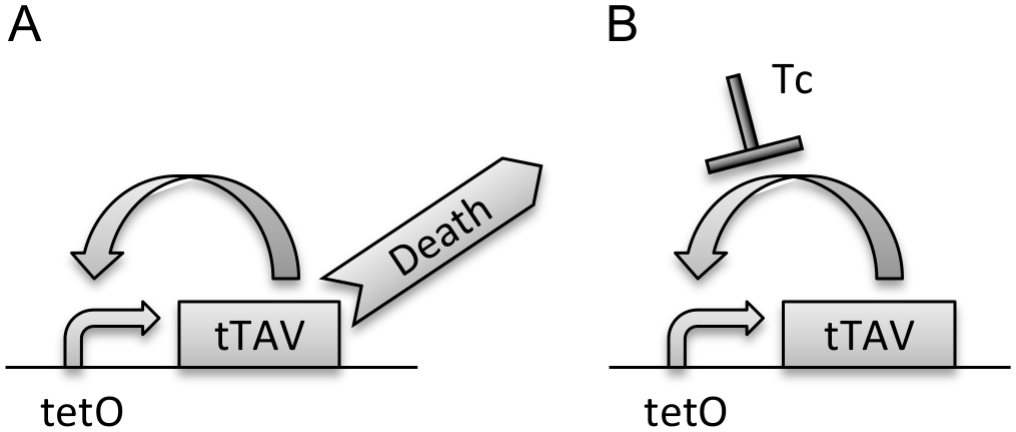
Tetracycline-repressible positive feedback loop in OX513A. In the absence of tetracycline (A) tTAV is expressed and causes mortality. In the presence of tetracycline (TC) (B) tetracycline binds to and represses the tTAV and allows development to adulthood (adapted from [35]).

To enable the breeding of OX513A individuals in the laboratory, the self-limiting phenotype of the transgene is repressed by the provision of tetracycline in the larval rearing water. In the absence of tetracycline, the transcriptional transactivator, tTAV (a synthetic sequence comprising a tetracycline binding domain and transactivator), binds to its DNA binding site, tetO (tetracycline operator), to drive high levels of expression of tTAV [4] (Fig 1A). The production of tTAV results in over expression of this protein via a positive feedback loop; intracellular accumulation of tTAV becomes cytotoxic, eventually leading to the organism´s death [4, 7]. However, if OX513A larvae are reared in water containing tetracycline at sufficient concentrations, the tetracycline binds to tTAV causing a conformational change, preventing its binding to tetO and stopping establishment of the self-limiting positive feedback loop (Fig 1B) [8]. For this system to work as intended, the self-limiting phenotype must be sufficiently repressed by tetracycline provided during rearing in contained conditions. Conversely, for field effectiveness, the self-limiting phenotype must not be substantially repressed by tetracycline at doses found in the larval habitats of this mosquito. Here we look in detail at the interaction of OX513A with tetracycline(s) and the impact on phenotype.

Tetracyclines are a family of broad-spectrum antibiotics. They are well known in human therapy and, owing to their low cost, are used in animal production as therapeutics, prophylactics and growth promoters, although many countries have banned this use [9, 10]. Agricultural use in particular can result in significant quantities being excreted, ending up in sewage systems or in manure [10]. However, although tetracyclines can be adsorbed to certain soils [11], they are highly susceptible to photolysis and hydrolysis giving some tetracyclines half-lives of only a few hours, limiting their environmental load [12]. Fortunately, there is considerable interest in antibiotic load in the environment and numerous surveys have been conducted: for tetracyclines these focus primarily on tetracycline, oxytetracycline, chlortetracycline and doxycycline. A survey of the literature found maximum reported concentrations from field sites around the world as follows; tetracycline 0.096 ng mL^-1^ to 1.3 ng mL^-1^ (e.g. [13–17]) chlortetracycline 0.04 ng mL^-1^ to 0.97 ng mL^-1^ [18, 19], oxytetracycline 0.7 ng mL^-1^ to 1.34 ng mL^-1^ [20, 21] and doxycycline 0.07 ng mL^-1^ to 0.4 ng mL^-1^ [14, 20].

These data have largely been generated from examination of tetracycline compound levels in input and output flows from wastewater treatment plants, where they are expected to reach particularly high concentrations as a result of excretion during therapeutic use, along with the testing of the efficiency of removal of tetracyclines during the treatment of the wastewater. However these environments are not typical *Ae*. *aegypti* larval habitats which include artificial containers such as used car tyres, flower vases, water storage vessels and discarded materials in the domestic and peri-domestic environment. Reports have also been made of *Ae*. *aegypti* breeding in septic tanks [22] and brackish waters [23], especially where covers are broken or cracked but these are regarded as unusual.

Given that tetracyclines are present in the environment, albeit at low levels, it is important to understand the possible routes of exposure of OX513A individuals to these tetracyclines and the impact that such exposure could have on its phenotype, especially on the expression of the self-limiting trait. We have therefore identified potential routes of exposure of OX513A to environmental sources of tetracyclines; these include larval breeding sites and human and veterinary patients, where potential environmental concentrations of tetracyclines have been explored and have also investigated the impact of such exposure at relevant concentrations on the phenotype of OX513A.

## Methods

### Mosquito strains

OX513A is a tetracycline repressible bi-sex lethal strain of *Aedes aegypti* [24].

The wild type (WT) strain used is a non-transgenic version of the genetic background of OX513A.

### Insect rearing

All insects were reared at 27°C [+/- 1°C], 70% [+/- 10%] relative humidity, 12h: 12h light: dark cycle. Unless stated otherwise, five cohorts of 200 larvae for each treatment were reared at 1 larva mL^-1^ in 16 oz pots (Robertson Packaging, UK SICC65) and fed a standard regimen of finely ground Tetramin fish flakes (Tetra GmbH, Germany). Live pupae were counted and placed into cages (15 cm x 15 cm x 15 cm, Bugdorm-Megaview, Taiwan). Dead larvae and dead pupae were counted and discarded. Adults were provided with 10% sucrose solution *ad libitum*. Adult cages were assessed three days after the last pupa was added. Assessment included counting the total number of dead pupae, non-viable adults (dead adults on the water, dead adults on the floor of the cage and non-flying adults) and functional adults (flying adults).

### Homozygous outcrosses

OX513A homozygous larvae were reared in the presence of 30 μg mL^-1^ chlortetracycline (the standard tetracycline supplement used in rearing); WT larvae were reared at a similar density in the absence of tetracycline. Larvae were fed *ad libitum*. Unless otherwise stated, OX513A males were outcrossed to WT females. Adults were blood fed using defibrinated horse blood (TCS Biosciences Ltd., UK). Heterozygous eggs were collected and used for the following experiments.

### Dose response of OX513A to tetracycline and its analogues

An initial tetracycline dose response curve was produced using chlortetracycline hydrochloride (Sigma-Aldrich, UK). Following this, tetracycline, oxytetracycline and doxycycline were tested (Sigma-Aldrich, UK). In all of these experiments heterozygous OX513A larvae were reared in the presence of different concentrations of chlortetracycline, tetracycline, oxytetracycline or doxycycline. As a control, OX513A were also reared in parallel in the absence of tetracycline or its analogues. Preliminary experiments were used to establish the appropriate dose range for each compound. Raw data are presented in S1 Table.

### Changes in chlortetracycline concentration during mass rearing

**Fig 2:**
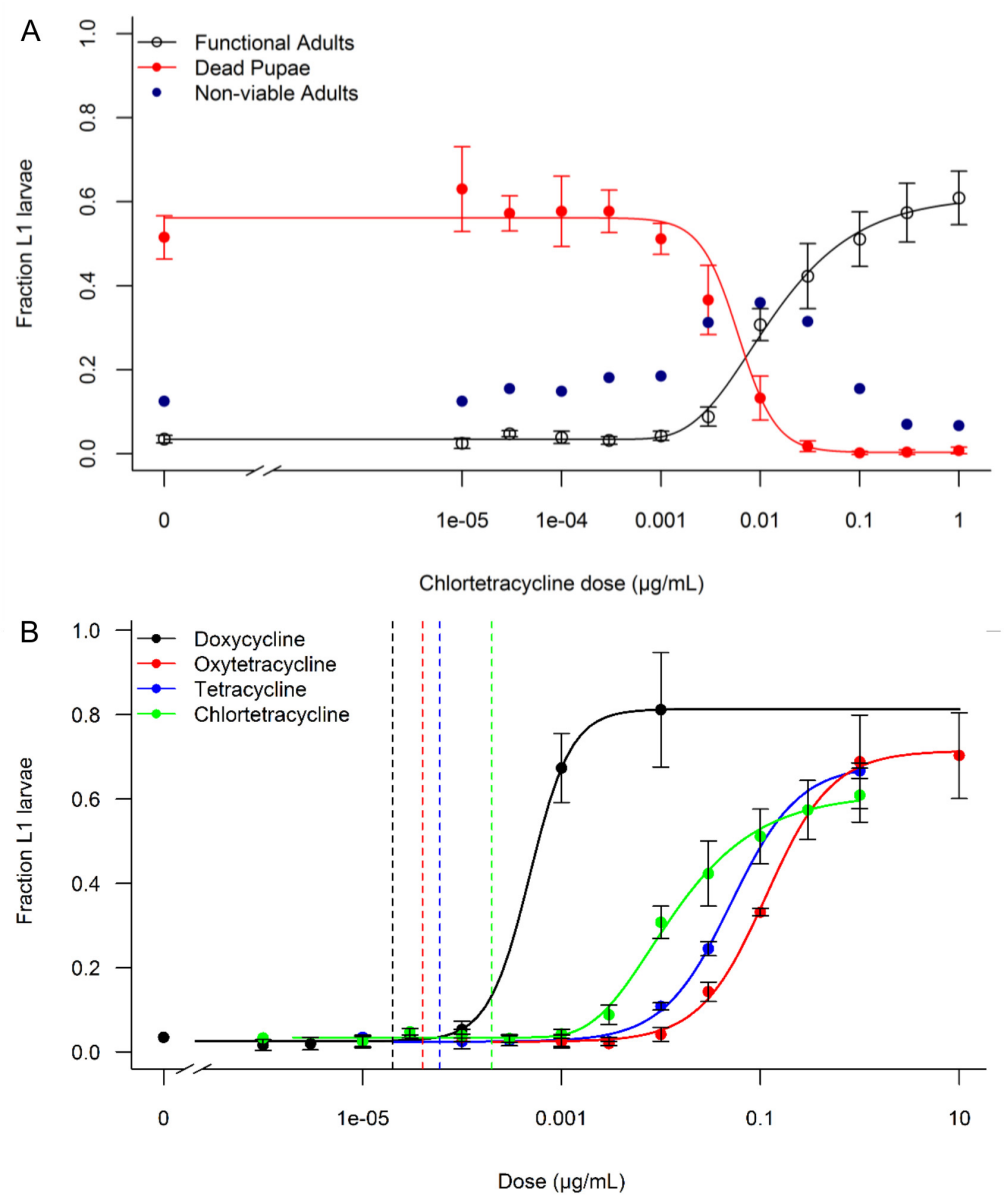
Dose response of OX513A to tetracycline and its analogues. A) Chlortetracycline dose response. Data shown for each of the three developmental outcomes for first instar larvae (L1) is shown: mortality as pupae, eclosion as non-viable adult, eclosion as functional (flying) adult. B) Dose response for four tetracyclines. Proportion of L1 surviving to functional adult data is shown after rearing on various concentrations of each tetracycline. Solid lines indicate the best-fit model; see also Table 2 for EC_50_ calculated from this model. Dashed vertical lines represent mean concentrations of each analogue (in the same colour as the lines themselves) found in environmental bodies of water [15, 20, 36–40]. If reported values were below the limit of quantification (LOQ), the LOQ value was used to populate the mean. Error bars represent the 95% confidence intervals.

Samples were collected at life stages between eggs and pupae (Fig 2). For each sampling point, 5 repeats of 0.5 g of the relevant mosquito life-stage were collected along with 5 repeats of 1.5 mL of the associated rearing water. To sample L1/L2 larvae (1 day post hatching) OX513A eggs were hatched and the larvae reared for 24 hours in the absence of tetracycline after which the larvae and the rearing water samples were collected. For samples of L3, L3/L4 and pupae, OX513A eggs were hatched and the larvae reared at the mass rearing density of 3 larvae mL^-1^ in 1 L of water supplemented on day one post hatching with chlortetracycline (Sigma-Aldrich, UK) to a final concentration of 30 μg mL^-1^. The samples were stored at -20°C before being shipped on dry ice to CEM Analytical Services (Bracknell, UK) for analysis.

Samples were analysed in accordance with the Association of Analytical Communities (AOAC) methodology for analysis of tetracycline in edible animal tissues (AOAC method 995.09) with the modification of LC-MS/MS (liquid chromatography with positive-ion electro-spray tandem mass spectrometry) for the final quantitation rather than UV, along with a recovery sample. Residues of chlortetracycline were extracted from the specimens by homogenizing in EDTA solution (pH 4). Two further extractions were combined with the first extract, filtered and the sample further purified using a C18, solid phase extraction cartridge. After conditioning the cartridge, the total volume of extract was percolated through the cartridge. After washing, the chlortetracycline was eluted using a methanolic oxalic acid solution and the final extract diluted with water. The samples were analysed by LC-MS/MS, using the transition 479.2 > 444.1 amu with a 0.1% formic acid in water/methanol mobile phase and a 250 x 4.6 mm C8, 5 μm column. Raw data are presented in S2 Table.

### Concentration of tetracyclines in environmental water samples

#### Sampling site selection

Water sampling points were selected based on the possible presence of tetracyclines in surface waters, or presence of *Ae*. *aegypti* larvae. Tetracyclines are found in surface water bodies impacted by sewage input or veterinary activity, especially intensive poultry and fish production. Considering this information, surface water samples were collected from three different sewage impacted creeks in Campinas, São Paulo State, Brazil (Pinheiros Creek, Anhumas Creek and Piçarrão Creek), one private fish production lake and a stream located near a poultry CAFO (Concentrated Animal Feeding Operation). Water samples were also acquired from known *Ae*. *aegypti* breeding sites located in an industrial equipment facility in Campinas. Samples were collected on 27^th^ November 2013, in the rainy season. Water was sampled from six different types of container, mainly refuse and discarded materials (images in S1 Fig), all of which contained larvae at the time of sampling. In addition to these environmental samples, rain water and tap water samples were collected in Campinas and in Itu, São Paulo, Brazil. For all sampling points, 1 L water samples were collected in triplicate, resulting in 45 samples. GPS coordinates for sampling points are provided in S3 Table.

#### Sampling procedure

The sampling of water was carried out using amber glass bottles (1 L) previously cleaned in the laboratory and rinsed with the sample water on site. The bottles were wrapped in aluminum foil and transported on ice to the laboratory. All samples were immediately submitted to extraction and clean up procedures.

#### Chemical analysis

Each sample was tested for the three tetracyclines most commonly reported in the environment due to their extensive use in agriculture: tetracycline, oxytetracycline and chlortetracycline.

Raw samples (1 L) were acidified to pH 3 using HCl, 500 mg of Na_2_EDTA were added and samples were filtered through 0.45 μm cellulose acetate membranes. Tetracyclines were extracted from water samples using a SPE procedure in a lab-made system [25]. OASIS HLB cartridges (500 mg, Waters) were used as sorbent and were conditioned with 6 mL of methanol, 6 mL of deionized water and 6 mL of 0.5 g L^-1^ Na_2_EDTA solution (pH 3). After sample elution, cartridges were dried under nitrogen flow for 20 minutes and tetracyclines were extracted using 6 mL of methanol. Solvent was carefully evaporated to dryness with a gentle flow of nitrogen and the target compounds were resuspended to a final volume of 1.0 mL in a 0.1% formic acid solution in water:methanol, 90:10 (v/v).

Tetracycline compound determination was performed by LC-MS/MS (Agilent 6410) using a Zorbax SB-C18 column (2.1 x 30 mm, 3.5 μm particle size) and deionized water (A) and methanol (B) containing 0.1% formic acid as mobile phase solvents at a flow rate of 0.3 mL min^-1^. The gradient composition was 10% B for 1 min, followed by an increase of B to 100% in 7 min. This composition was held for 1 min, when the initial conditions were re-established and maintained for 5 min before the next analysis. Tetracyclines were ionized using an electrospray source (ESI) in positive mode. Ultrapure nitrogen (99.995%) was used as drying gas (350°C at a flow rate of 10 L min^-1^) and in the collision cell. Nebulizer pressure was set at 55 psi and a capillary voltage of 2000 V was used. Multiple reaction monitoring (MRM) parameters for each of the tetracyclines are shown in S4 Table.

### Effects of adult ingestion of chlortetracycline on their OX513A progeny

OX513A homozygous larvae were reared at <1 larvae mL^-1^ in the presence of 30 μg mL^-1^ chlortetracycline and WT larvae were reared in the absence of tetracycline. Adults were placed into cages as follows; OX513A♂ crossed to WT♀ and OX513A♀ crossed to WT♂.

Adult cages were assigned to one of three chlortetracycline treatments; 50 μg mL^-1^ chlortetracycline sugar water and 50 μg mL^-1^ chlortetracycline blood meal, 100 μg mL^-1^ chlortetracycline sugar water and 100 μg mL^-1^ chlortetracycline blood meal, or chlortetracycline free sugar water and chlortetracycline free blood meal (not performed for OX513A♀ crossed to WT♂) (Table 1). Each chlortetracycline treatment had three repeats for each adult cross combination. Eggs were collected. Chlortetracycline concentrations were chosen to reflect levels well above the maximum recorded plasma levels following a therapeutic treatment with tetracyclines [26, 27].

**Table 1:** Experimental design of OX513A chlortetracycline ingestion. Parental crosses of OX513A homozygotes were crossed to wild type (WT). These adults were provided with blood and sugar water supplemented with chlortetracycline (TC) at three concentrations; 0 μg mL^-1^, 50 μg mL^-1^ or 100 μg mL^-1^. Progeny were reared in cohorts of 200 individuals in the presence or absence of chlortetracycline.

		Parental cross			Progeny
Group	Description	Male	Female	TC Blood and Sugar (μg mL^-1^)	TC
A	No TC control	OX513A	WT	0	NO
B	Experimental	OX513A	WT	50	NO
C	Experimental	WT	OX513A	50	NO
D	Experimental	OX513A	WT	100	NO
E	Experimental	WT	OX513A	100	NO
F	Rearing control	OX513A	WT	0	YES
G	Rearing control	OX513A	WT	50	YES

The heterozygous progeny from each treatment were reared according to the insect rearing method described above with six cohorts set for each group to assess the effect of parental chlortetracycline ingestion. Raw data are presented in S5 Table.

### Statistical analyses

Data were analysed using the RStudio software package version 0.97.318 (RStudio, USA). Normality of data was tested using the Shapiro-Wilk method.

Bioaccumulation of chlortetracycline in the biological materials was shown to be not normally distributed and therefore analysed using Wilcoxon Rank Sum.

Dose response data was analysed for significant differences between concentrations using either a Student’s *t*-test if normally distributed or Wilcoxon Rank Sum if not normally distributed. The data was modelled using the Weibull model for chlortetracycline and a Log-linear model for the remaining antibiotics. These models were used to calculate the EC_50_ (Effective Concentration_50_) with 95% confidence intervals.

Chlortetracycline ingestion data were analysed using ANOVA for normally distributed data or a Kruskal-Wallis test for non-normally distributed data. Post-hoc analysis was carried out using the Nemenyi test, using the Coin and Multcomp R packages. For the on-tetracycline reared controls, differences in functional adults were tested using the Wilcoxon Rank Sum test.

## Results

### Dose response of OX513A to tetracycline and its analogues

OX513A larvae reared at chlortetracycline concentrations at or below 1 ng mL^-1^ did not give rise to a significantly greater percentage of functional adults than larvae reared in the absence of tetracycline (0 μg mL^-1^) (*t*(18) = -1.36, *p* = 0.19). Functional adult numbers begin to deviate from the baseline when reared at 3 ng mL^-1^ (*t*(13) = -3.49 *p* = 0.004), therefore concentrations in excess of 1 ng mL^-1^ give rise to an increasing fraction of functional adults. Fig 2A shows the percentage of functional adults increasing with greater chlortetracycline concentrations, with the model predicting the maximum rescue plateau beginning to appear at 1 μg mL^-1^. This indicates that concentrations at or slightly above 1 μg mL^-1^ will give rise to the maximum percentage of functional adults.

For the other tetracycline compounds tested, concentrations at or below 3 ng mL^-1^ tetracycline, 10 ng mL^-1^ oxytetracycline and 0.1 ng mL^-1^ doxycycline, did not give rise to a significantly greater portion of functional adults than if reared in the absence of tetracycline (*Z* = 6.5, *p* = 0.48; *Z* = 3, *p* = 0.64; *Z* = 1.5, *p* = 0.27, respectively) (Fig 2B).

For all four tetracyclines, increasing tetracycline concentrations resulted in incremental shifts towards increased survivorship of OX513A individuals (Fig 2A and 2B). With reference to Fig 2A, as the chlortetracycline concentration increases to result in more functional adults than 0 μg mL^-1^ tetracycline, the sample population shifts towards a higher survivorship with an increasing fraction of non-viable adults and decreasing fraction of dead pupae. With further increases in the chlortetracycline concentration, the non-viable adult fraction drops and the population shifts to eclosing as functional adults. This demonstrates that increasing tetracycline concentrations have incremental rescue effects on OX513A resulting in an average increase in survivorship represented by an increased proportion of flying functional adults.

The EC_50_ values (half maximal effective concentration; the concentration which induces a response halfway between the baseline and maximum) shown in Table 2, demonstrate that doxycycline is the most effective analogue at rescuing the OX513A phenotype, being able to rescue individuals at a lower concentration than the other tetracyclines.

**Table 2:** EC_50_ of tetracycline and analogues at rescuing OX513A. Tetracycline and analogues were added to the larval rearing water at various concentrations and the EC_50_ (half maximal effective concentration) modelled. 95% confidence intervals are shown in parenthesis. Results are for functional (flying) adults.

Compound	EC_50_ ng mL^-1^ (95% C.I.)
Oxytetracycline	113 (89 to 138)
Tetracycline	50 (34 to 66)
Chlortetracycline	13 (9 to 16)
Doxycycline	0.48 (0.40 to 0.60)

### Changes in chlortetracycline concentration during mass rearing

**Fig 3:**
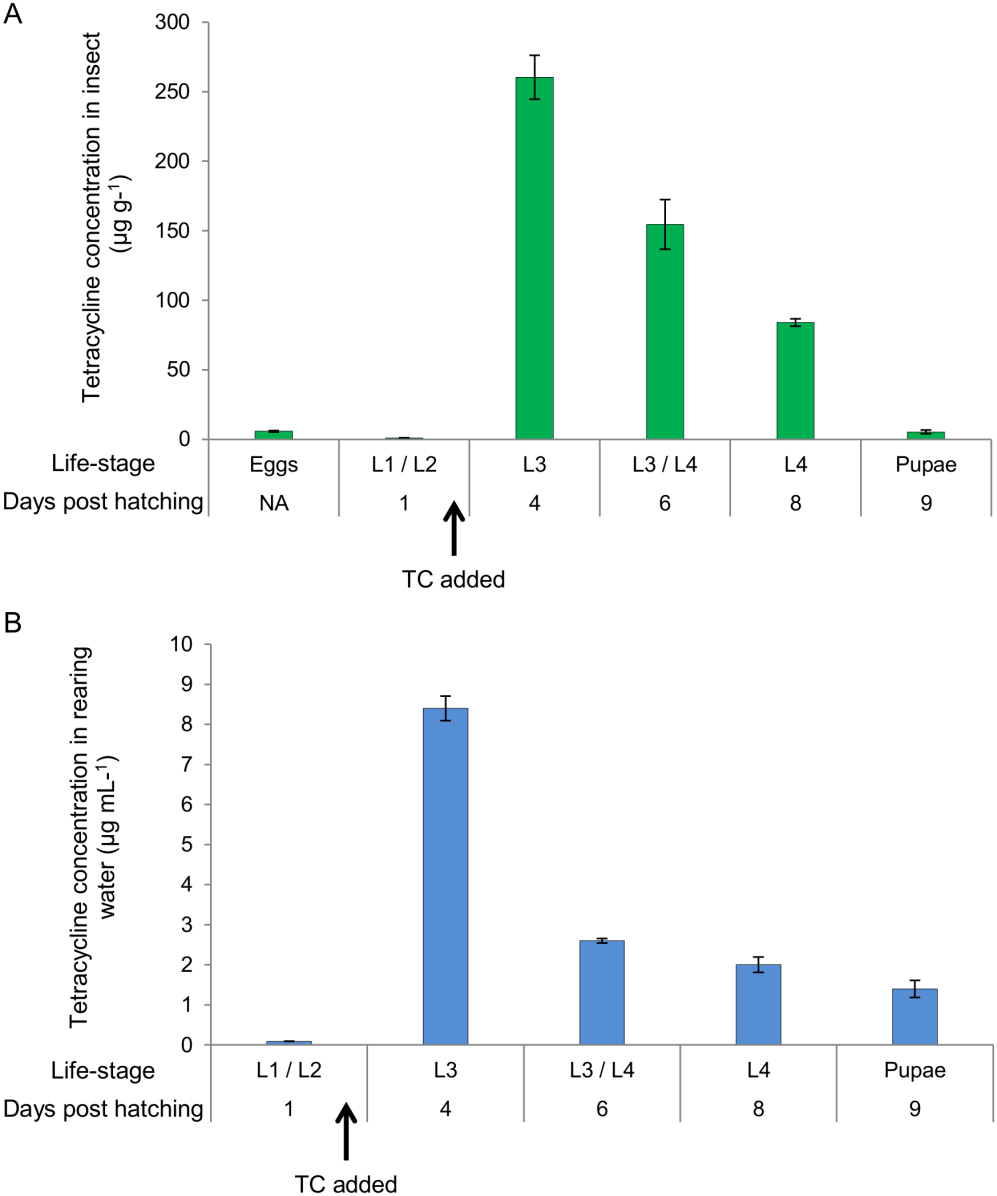
Life-stage analysis of the change in concentration of chlortetracycline. Chlortetracycline (TC) was added to the rearing water to a concentration of 30 μg ml^-1^ one day post hatching, immediately after L1 / L2 sample was taken as indicated. At each sampling point, samples of the rearing water and relevant insect life-stage (eggs/larvae (L1-L4)/pupae were collected. Tetracycline concentration was determined in A) OX513A mosquito larvae and pupae and B) in the associated rearing water. Life-stage is indicated along with days post hatching (1–9). Error bars represent SEM.

Quantification of the changes in chlortetracycline within OX513A over the duration of rearing demonstrates that chlortetracycline is bio-accumulated to a concentration of 260 μg g^-1^eight times greater than the concentration initially present in the rearing water (30 μg mL^-1^) (Fig 3). The peak in bioaccumulation appeared at four days post hatching, when the larvae had reached the third instar (L3). After this point, the concentration of chlortetracycline in the biological samples decreased so that by the pupal stage there was no significant difference between the concentration in the pupae compared to the eggs or L1 larvae, which is before exposure to tetracycline (*Z* = 29.5 *p* = 0.62). Samples of male and female pupae were analysed separately for differences in bioaccumulation but no significant difference was found (*t*(8) = 0.16, *p* = 0.88); combined data are therefore presented in Fig 3A.

The analysis of the associated larval rearing water indicates that the concentration of chlortetracycline decreased rapidly over the first four days of rearing from the starting concentration of 30 μg mL^-1^ to 8.4 μg mL^-1^ (Fig 3B). This decrease correlates with the bioaccumulation of the chlortetracycline seen in the larvae, as well as some of the chlortetracycline likely being degraded by hydrolysis and photolysis. The concentration in the rearing water continued to decrease over the remaining rearing days until only 1.4 μg mL^-1^ remained by the initiation of pupation. This is 21 times lower than the starting chlortetracycline concentration.

### Concentration of tetracyclines in environmental water samples

Water samples were collected from *Ae*. *aegypti* breeding sites to determine the environmental concentrations of tetracyclines that OX513A larvae are likely to encounter in the field. Water sampling sites were selected based on their potential to contain high concentrations of tetracyclines (close to sewage plants or intensive livestock operations) or for being an *Ae*. *aegypti* larval habitat, as determined by the presence of larvae. The concentration of each tetracycline tested; tetracycline, oxytetracycline and chlortetracycline, was below the limit of quantification for each of the field samples. The limit of detection was 1.0 pg mL^-1^ for tetracycline and chlortetracycline and 2.5 pg mL^-1^ for oxytetracycline.

### Effects of adult ingestion of chlortetracycline on their OX513A progeny

**Fig 4:**
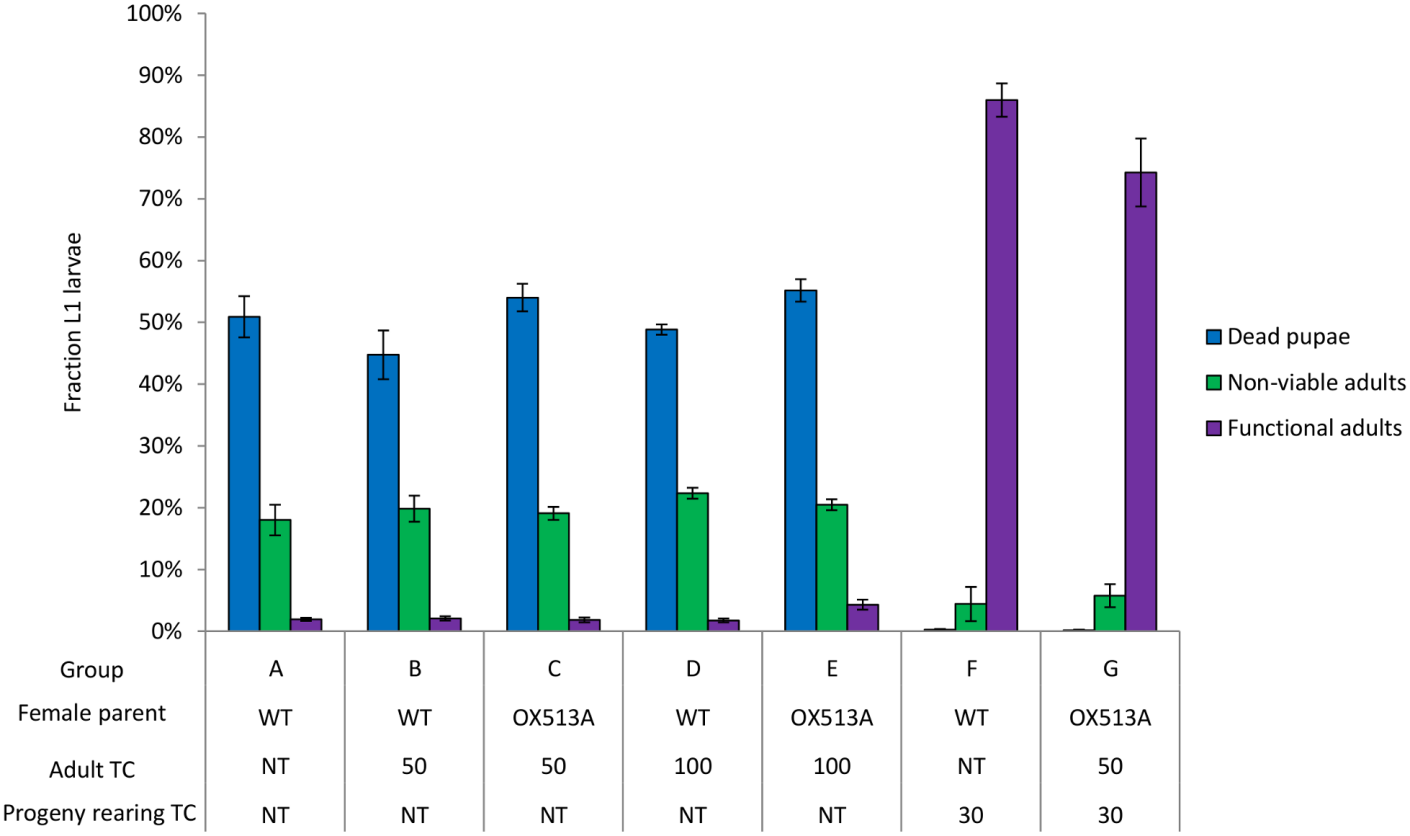
Phenotype of OX513A progeny from parents provided with tetracycline. OX513A adults crossed to wild type (WT) were provided with blood and sugar water supplemented with chlortetracycline. Their progeny (data presented here) were reared in the presence (control) or absence of chlortetracycline to determine the effect of parental ingestion of tetracycline. Error bars represent SEM. A to G represents groups in Table 1. Adult TC is the concentration of chlortetracycline in the blood and sugar meals of the parents (μg mL^-1^). NT is no tetracycline administered. Progeny rearing TC is the concentration of chlortetracycline added to the rearing water (μg mL^-1^).

We investigated whether parental exposure to chlortetracycline could influence the survival of heterozygous OX513A offspring. This might occur if tetracycline-fed females loaded tetracycline in their eggs, for example. To provide maximal exposure to chlortetracycline, in all experimental groups both blood and sugar water were supplemented with chlortetracycline to 50 μg mL^-1^ or 100 μg mL^-1^ (Table 1). No significant difference was observed between the progeny of these groups (groups A to E) and those of the controls not provided with tetracycline (group A) in terms of the fraction of dead pupae (*F*(4,25) = 2.44, *p* = 0.07), non-viable adults (*F*(4,25) = 1.00 *p* = 0.43) or functional adults (*H*(4) = 9.93, *p* = 0.04, followed by Nemenyi post-hoc analysis shows non-tetracycline-loaded control (A) vs experimental groups (B, C, D and E) *p* = ≥0.05.). Where larvae were provided with chlortetracycline, provision of chlortetracycline to the parents did not further increase survival (*Z* = 29, *p* = 0.09) (Fig 4, group F compared to group G).

## Discussion

Owing to their use as human and veterinary therapeutics and prophylactics, tetracyclines are known to be present in the environment, albeit at low levels as they are subject to photolysis, hydrolysis and adsorption [11, 12]. In the absence of tetracycline, the tTAV gene in the strain OX513A leads to a self-limiting phenotype, which can be rescued to near-normal viability by sufficient levels of a suitable tetracycline [4]. For OX513A to be used as an effective vector control tool, it is important to understand the concentrations of tetracyclines which will allow an increase in survival of OX513A relative to tetracycline-free conditions, and the possibility, if any, of exposure of *Ae*. *aegypti* to such concentrations in the field.

Given that tetracyclines could be present in the environments where OX513A is used to supress wild *Ae*. *aegypti* populations, it is important to know the lowest concentrations of tetracyclines that would allow a greater than nominal fraction of functional adults to survive, as well as understanding the consequences if larvae were to encounter tetracyclines in the environment in terms of the numbers of potential functional females expected to emerge. This will help to inform on the efficiency and speed of suppression of the *Ae*. *aegypti* population. Models indicate that to have a significant impact on programme effectiveness, average OX513A heterozygote fitness would have to increase to about 10% of WT, which would be equivalent to 10% survival of L1s to functional adults, assuming such adults were fully fit [4]. We tested the effects of four tetracyclines reported to be found in the environment from their use in human therapy or agriculture on larval survival. We found that concentrations at or below 3 ng mL^-1^ tetracycline, 1 ng mL^-1^ chlortetracycline, 10 ng mL^-1^ oxytetracycline and 0.1 ng mL^-1^ doxycycline gave no significant increase in the survivorship of OX513A larvae, i.e. did not increase the proportion of functional adults. Full rescue of the OX513A individuals (the maximum number surviving to functional adults) was also shown in this data to require tetracycline compound concentrations that were 746 to 2500 times greater than the highest concentrations we found reported in the literature for environmental tetracyclines.

In surveying the literature we found a few instances of reported environmental concentrations of doxycycline above the concentration which would allow a greater than the nominal fraction of OX513A larvae to develop to functional adults. However, such studies are often interested in the impact of treatment and removal of antibiotics in waste-water treatment plants (e.g.[14, 20]) and their associated rivers, and find the maximal tetracycline compound concentrations to be in the sewage input to treatment plants. These environments are not the typical larval habitats of *Ae*. *aegypti*. This species is a ‘container breeding mosquito’; its preferred breeding sites are refuse, flower vases, water storage containers and other peri-domestic sites. Immature stages are found in clean, still water, not flowing river systems and are rarely found in collections of water in the ground such as earth drains [28–30]. Some reports have suggested that *Ae*. *aegypti* can breed in septic tanks, usually where they are cracked or broken [22, 31], but this tends to be in the clear water at the top of the tank whereas tetracyclines tend to bind to the sediments located at the bottom of the tanks [16, 32] and are expected to represent a minority of breeding sites. As a consequence of the lack of relevant field data, we undertook field sampling of actual *Ae*. *aegypti* larval habitats and analysed the concentration of three tetracycline compounds. We found that for the tetracyclines assessed, the concentration was below the level of detection, which was in turn below the concentration necessary to allow any increase in survivorship of OX513A. Taken together, these data indicate that levels of environmental tetracyclines are unlikely to lead to a significant increase in the survival of the progeny of released OX513A males.

For a suppression programme, OX513A males will be reared in a factory in the presence of a tetracycline compound either in, or close to, the area of male release. It is therefore important to understand how the mass production of OX513A males, which requires the use of tetracyclines, could affect the environmental concentrations of tetracyclines. Currently larvae are mass reared in the presence of excess chlortetracycline (30 μg mL^-1^), but it was previously unknown how much of the chlortetracycline the larvae accumulated over the course of development and how much remained at the end of a rearing cycle. Here we have found that the OX513A larvae bioaccumulate the chlortetracycline to over 8 times the initial starting concentration. As a consequence of this process, and likely owing to some degree of photolysis and hydrolysis, the concentration of chlortetracycline in the rearing water at the end of the rearing cycle is 21 times lower than at the start. This is an important consideration in respect of potential effluent from mass rearing production. We estimate from these data that the spent water from field rearing for an operational suppression programme, for example one aimed to treat a human population of 10 million, would contain approximately 136 to 291 kg chlortetracycline per annum. Putting this figure in context, it is estimated that the USA in 2011 used over 5 million kg of tetracyclines in food-producing animals [33] and a further 113,832 kg in human therapy [34]. Consequently the estimated waste chlortetracycline from the production of OX513A males for a suppression programme constitutes a minute fraction of the total tetracyclines used in just one country.

Finally we investigated the hypothesis that the very small percentage of co-released OX513A adult females [5] exposed to chlortetracycline in their diet could pass sufficient quantities through their eggs to allow their progeny an increased level of survival. Tetracyclines are administered in human therapy and as *Ae*. *aegypti* are highly anthropophilic, it could be envisaged that females, after a blood meal from a human (or rarely an animal) taking a therapeutic course of tetracyclines, could ‘pre-load’ their progeny with enough tetracycline compound to allow an increase in survival. In mammals the concentration of tetracycline compounds in the blood usually reaches a peak 2 to 6 hours following an oral or injected dose, and then gradually declines due to the body’s metabolic activity [26]. In both humans and livestock, the peak concentration of tetracyclines in blood (plasma) following a standard therapeutic dose normally remains below 10 μg ml^-1^ [26, 27]. We found that feeding adult females 50 μg mL^-1^ or 100 μg mL^-1^ chlortetracycline in both blood and sugar water did not lead to increased survival of their progeny. The highest dose of chlortetracycline used in this study was 10-fold higher than the normal concentration found in the blood of humans or animals receiving usual therapeutic doses of tetracyclines, and 5-fold higher than the highest dose reported from any animal blood [27]. These data therefore disprove the hypothesis that a female can deliver enough tetracycline to her progeny to allow increased survival rates through biting a human or animal on a normal therapeutic dose of tetracycline.

The results presented here analyse the interaction of tetracycline and its analogues with the OX513A phenotype and explore different scenarios by which OX513A individuals may be exposed to tetracyclines in the environment. Collectively, these data demonstrate that the low levels of tetracycline or its analogues likely to be encountered in the environment will not impact the efficacy or safety of using OX513A to control *Ae*. *aegypti*.

## Supporting Information

S1 Fig *Aedes aegypti* larval breeding sites where water samples were collected for the environmental tetracycline concentration analysis. These containers are typical breeding sites of *Ae*. *aegypti* and were all positive for *Ae*. *aegypti* larvae when the water samples were collected.
(TIF)

S1 Table Dose response of OX513A to tetracycline and its analogues. Raw data from each analogue (tetracycline, chlortetracycline, doxycycline and oxytetracycline) is presented in a separate table.
(DOCX)

S2 Table Chlortetracycline concentration in biological samples and in the associated rearing water of OX513A mass production. Raw data.
(DOCX)

S3 Table GPS coordinates of water sampling points in Brazil. (DOCX)

S4 Table Retention time, MRM transitions, fragmentor voltage and collision energy (CE) for each tetracycline analyte; oxytetracycline (OTC), tetracycline (TC) and chlortetracycline (CTC).
(DOCX)

S5 Table Effects of adult ingestion of chlortetracycline on OX513A progeny. Raw data. Refer to Table 1 for information on treatment.
(DOCX)
