# Supplementary figures and images for: Assessment of the Impact of Potential Tetracycline Exposure on the Phenotype of Aedes aegypti OX513A: Implications for Field Use
Source: PLoS Negl Trop Dis. 2015 Aug 13;9(8):e0003999. doi: 10.1371/journal.pntd.0003999 (PMC4535858; doi:10.1371/journal.pntd.0003999)

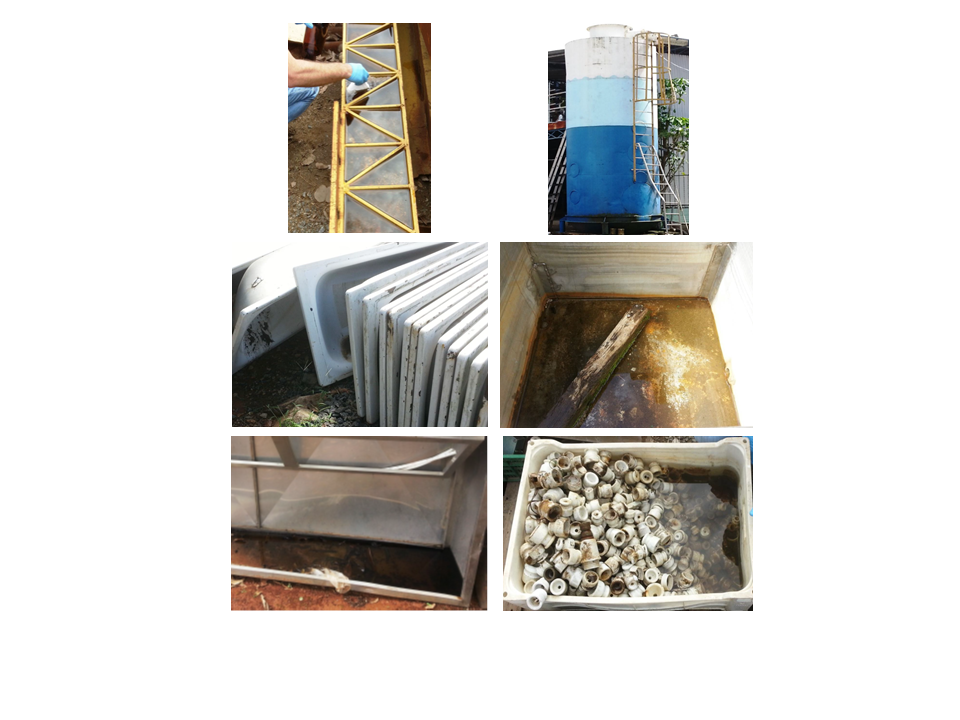

Supplement: S1 Fig — These containers are typical breeding sites of Ae. aegypti and were all positive for Ae. aegypti larvae when the water samples were collected. (TIF) [file pntd.0003999.s001.tif]
