# Supplementary material for: Assessment of the Impact of Potential Tetracycline Exposure on the Phenotype of Aedes aegypti OX513A: Implications for Field Use
Source: PLoS Negl Trop Dis. 2015 Aug 13;9(8):e0003999. doi: 10.1371/journal.pntd.0003999 (PMC4535858; doi:10.1371/journal.pntd.0003999)
Supplement: S1 Table — Raw data from each analogue (tetracycline, chlortetracycline, doxycycline and oxytetracycline) is presented in a separate table. (DOCX) [file pntd.0003999.s002.docx]

**S1 Table. Dose response of OX513A to tetracycline and its analogues.** Raw data from each analogue (tetracycline, chlortetracycline, doxycycline and oxytetracycline) are presented in a separate table.

| Chlortetracycline concentration | Rep | Dead pupae | Dead adults on water | Dead adults on cage | Non flying adults | Flying adults |
| --- | --- | --- | --- | --- | --- | --- |
|  |  |  |  |  |  |  |
|  |  |  |  |  |  |  |
| 1 µg/mL | 1 | 0 | 26 | 4 | 0 | 122 |
|  | 2 | 0 | 3 | 0 | 3 | 131 |
|  | 3 | 3 | 10 | 0 | 2 | 139 |
|  | 4 | 4 | 10 | 1 | 3 | 116 |
|  | 5 | 1 | 2 | 2 | 1 | 101 |
| 0.3 µg/mL | 1 | 0 | 9 | 1 | 1 | 134 |
|  | 2 | 0 | 2 | 0 | 5 | 115 |
|  | 3 | 1 | 24 | 1 | 3 | 111 |
|  | 4 | 0 | 16 | 1 | 1 | 91 |
|  | 5 | 3 | 5 | 1 | 0 | 123 |
| 0.1 µg/mL | 1 | 0 | 15 | 0 | 0 | 76 |
|  | 2 | 0 | 20 | 0 | 9 | 106 |
|  | 3 | 2 | 22 | 1 | 8 | 112 |
|  | 4 | 0 | 26 | 0 | 4 | 110 |
|  | 5 | 0 | 40 | 2 | 8 | 107 |
| 30 ng/mL | 1 | 3 | 50 | 0 | 12 | 84 |
|  | 2 | 1 | 43 | 0 | 16 | 65 |
|  | 3 | 1 | 56 | 2 | 5 | 73 |
|  | 4 | 5 | 38 | 2 | 8 | 111 |
|  | 5 | 8 | 65 | 11 | 7 | 90 |
| 10 ng/mL | 1 | 7 | 37 | 8 | 17 | 52 |
|  | 2 | 13 | 66 | 6 | 11 | 57 |
|  | 3 | 16 | 53 | 3 | 13 | 61 |
|  | 4 | 8 | 65 | 1 | 15 | 41 |
|  | 5 | 21 | 73 | 3 | 3 | 54 |
|  | 1 | 54 | 43 | 4 | 14 | 76 |
|  | 2 | 46 | 58 | 22 | 2 | 52 |
|  | 3 | 45 | 49 | 12 | 11 | 75 |
|  | 4 | 23 | 47 | 10 | 10 | 75 |
|  | 5 | 32 | 51 | 3 | 10 | 72 |
| 3 ng/mL | 1 | 52 | 47 | 6 | 9 | 31 |
|  | 2 | 50 | 59 | 7 | 2 | 21 |
|  | 3 | 52 | 45 | 11 | 5 | 9 |
|  | 4 | 41 | 69 | 1 | 0 | 8 |
|  | 5 | 50 | 50 | 7 | 7 | 27 |
|  | 1 | 98 | 62 | 10 | 0 | 17 |
|  | 2 | 114 | 35 | 10 | 6 | 15 |
|  | 3 | 87 | 49 | 8 | 0 | 18 |
|  | 4 | 94 | 46 | 7 | 0 | 19 |
|  | 5 | 94 | 58 | 8 | 1 | 12 |
| 1 ng/mL | 1 | 97 | 33 | 9 | 3 | 13 |
|  | 2 | 106 | 26 | 1 | 4 | 14 |
|  | 3 | 83 | 39 | 2 | 1 | 7 |
|  | 4 | 103 | 35 | 4 | 0 | 7 |
|  | 5 | 98 | 32 | 3 | 3 | 11 |
|  | 1 | 114 | 24 | 1 | 0 | 4 |
|  | 2 | 90 | 37 | 9 | 1 | 5 |
|  | 3 | 104 | 35 | 4 | 1 | 10 |
|  | 4 | 126 | 31 | 4 | 0 | 10 |
|  | 5 | 102 | 23 | 4 | 1 | 5 |
| 300 pg/mL | 1 | 112 | 32 | 5 | 0 | 6 |
|  | 2 | 126 | 27 | 3 | 1 | 7 |
|  | 3 | 127 | 29 | 5 | 0 | 8 |
|  | 4 | 113 | 43 | 4 | 2 | 8 |
|  | 5 | 99 | 28 | 0 | 2 | 3 |
| 100 pg/mL | 1 | 109 | 16 | 0 | 1 | 3 |
|  | 2 | 146 | 21 | 5 | 0 | 11 |
|  | 3 | 108 | 30 | 8 | 0 | 7 |
|  | 4 | 95 | 36 | 4 | 0 | 11 |
|  | 5 | 119 | 24 | 4 | 0 | 7 |
| 30 pg/mL | 1 | 117 | 26 | 3 | 0 | 8 |
|  | 2 | 108 | 20 | 2 | 1 | 12 |
|  | 3 | 116 | 28 | 4 | 1 | 8 |
|  | 4 | 128 | 29 | 1 | 0 | 10 |
|  | 5 | 103 | 36 | 3 | 1 | 10 |
| 10 pg/mL | 1 | 123 | 19 | 1 | 0 | 5 |
|  | 2 | 132 | 11 | 4 | 0 | 2 |
|  | 3 | 162 | 27 | 4 | 0 | 9 |
|  | 4 | 104 | 32 | 2 | 1 | 3 |
|  | 5 | 109 | 21 | 1 | 2 | 6 |
| 0 | 1 | 132 | 18 | 2 | 1 | 6 |
|  | 2 | 100 | 29 | 1 | 0 | 8 |
|  | 3 | 117 | 26 | 3 | 0 | 9 |
|  | 4 | 108 | 33 | 3 | 1 | 11 |
|  | 5 | 93 | 40 | 5 | 1 | 11 |
|  | 1 | 87 | 11 | 1 | 0 | 3 |
|  | 2 | 107 | 17 | 3 | 0 | 6 |
|  | 3 | 90 | 22 | 1 | 0 | 6 |
|  | 4 | 73 | 14 | 3 | 0 | 4 |
|  | 5 | 96 | 13 | 2 | 0 | 3 |

| Doxycycline concentration | Rep | Dead pupae | Dead adults on the water | Dead adults in the cage | Non-flying adults | Flying adults |
| --- | --- | --- | --- | --- | --- | --- |
| 1 pg/mL | 1 | 90 | 33 | 2 | 0 | 2 |
|  | 2 | 84 | 36 | 4 | 0 | 2 |
|  | 3 | 99 | 42 | 2 | 0 | 6 |
| 3 pg/mL | 1 | 91 | 24 | 1 | 0 | 5 |
|  | 2 | 88 | 22 | 4 | 0 | 1 |
|  | 3 | 92 | 45 | 2 | 1 | 6 |
| 10 pg/mL | 1 | 99 | 33 | 0 | 0 | 2 |
|  | 2 | 80 | 43 | 2 | 1 | 7 |
|  | 3 | 100 | 29 | 4 | 0 | 6 |
| 30 pg/mL | 1 | 63 | 32 | 3 | 0 | 8 |
|  | 2 | 71 | 40 | 1 | 0 | 7 |
|  | 3 | 75 | 27 | 1 | 0 | 7 |
| 100 pg/mL | 1 | 51 | 53 | 8 | 5 | 14 |
|  | 2 | 81 | 53 | 7 | 0 | 11 |
|  | 3 | 82 | 57 | 9 | 1 | 7 |
| 1 ng/mL | 1 | 1 | 19 | 1 | 27 | 143 |
|  | 2 | 1 | 28 | 0 | 30 | 118 |
|  | 3 | 2 | 41 | 0 | 15 | 143 |
| 1µg/mL | 1 | 1 | 2 | 3 | 0 | 168 |
|  | 2 | 2 | 1 | 1 | 0 | 183 |
|  | 3 | 2 | 1 | 0 | 0 | 136 |

| Oxytetracycline concentration | Rep | Dead pupae | Dead adults on the water | Dead adults in the cage | Non-flying adults | Flying adults |
| --- | --- | --- | --- | --- | --- | --- |
| 1 ng/mL | 1 | 103 | 35 | 5 | 0 | 2 |
|  | 2 | 77 | 41 | 2 | 0 | 6 |
|  | 3 | 77 | 28 | 2 | 0 | 7 |
| 3 ng/mL | 1 | 105 | 37 | 6 | 3 | 4 |
|  | 2 | 102 | 38 | 7 | 0 | 5 |
|  | 3 | 118 | 25 | 2 | 0 | 3 |
| 10 ng/mL | 1 | 98 | 41 | 3 | 0 | 10 |
|  | 2 | 98 | 38 | 8 | 0 | 10 |
|  | 3 | 111 | 37 | 7 | 0 | 5 |
| 30 ng/mL | 1 | 66 | 51 | 3 | 6 | 28 |
|  | 2 | 78 | 50 | 5 | 7 | 25 |
|  | 3 | 59 | 65 | 3 | 3 | 33 |
| 100 ng/mL | 1 | 27 | 50 | 3 | 4 | 65 |
|  | 2 | 27 | 43 | 1 | 4 | 66 |
|  | 3 | 37 | 46 | 5 | 6 | 68 |
| 1 µg/mL | 1 | 0 | 8 | 0 | 0 | 136 |
|  | 2 | 1 | 2 | 1 | 0 | 119 |
|  | 3 | 1 | 3 | 0 | 2 | 158 |
| 10 µg/mL | 1 | 0 | 1 | 0 | 0 | 123 |
|  | 2 | 0 | 3 | 1 | 0 | 140 |
|  | 3 | 1 | 1 | 0 | 1 | 159 |

| Tetracycline concentration | Rep | Dead pupae | Dead adults on the water | Dead adults in the cage | Non-flying adults | Flying adults |
| --- | --- | --- | --- | --- | --- | --- |
| 100 pg/mL | 1 | 109 | 31 | 7 | 0 | 2 |
|  | 2 | 106 | 44 | 3 | 1 | 8 |
|  | 3 | 110 | 31 | 4 | 0 | 5 |
| 300 pg/mL | 1 | 107 | 27 | 3 | 0 | 6 |
|  | 2 | 114 | 33 | 7 | 1 | 7 |
|  | 3 | 89 | 39 | 6 | 0 | 3 |
| 1 ng/mL | 1 | 78 | 25 | 1 | 0 | 5 |
|  | 2 | 70 | 38 | 0 | 0 | 8 |
|  | 3 | 109 | 29 | 3 | 0 | 3 |
| 3 ng/mL | 1 | 95 | 26 | 4 | 0 | 4 |
|  | 2 | 86 | 37 | 1 | 0 | 4 |
|  | 3 | 73 | 35 | 6 | 0 | 7 |
| 10 ng/mL | 1 | 78 | 50 | 2 | 0 | 22 |
|  | 2 | 70 | 43 | 5 | 1 | 20 |
|  | 3 | 47 | 37 | 7 | 6 | 23 |
| 30 ng/mL | 1 | 27 | 60 | 4 | 6 | 46 |
|  | 2 | 7 | 63 | 2 | 8 | 52 |
|  | 3 | 13 | 39 | 1 | 9 | 49 |
| 1 µg/mL | 1 | 2 | 1 | 0 | 0 | 131 |
|  | 2 | 0 | 5 | 0 | 0 | 137 |
|  | 3 | 1 | 10 | 1 | 3 | 132 |
