# Supplementary material for: Assessment of the Impact of Potential Tetracycline Exposure on the Phenotype of Aedes aegypti OX513A: Implications for Field Use
Source: PLoS Negl Trop Dis. 2015 Aug 13;9(8):e0003999. doi: 10.1371/journal.pntd.0003999 (PMC4535858; doi:10.1371/journal.pntd.0003999)
Supplement: S2 Table — Raw data. (DOCX) [file pntd.0003999.s003.docx]

**S2 Table Chlortetracycline concentration in biological samples and in the associated rearing water of OX513A mass production.** Raw data.

| Life stage sample | Residue in Sample (µg/g) |
| --- | --- |
| Spiked control | 5.28 |
| Spiked reagent | 0.42 |
| Eggs | 6.29 |
|  | 6.83 |
|  | 3.66 |
|  | 5.73 |
|  | 6.53 |
| L1/L2 Larvae | 1.60 |
|  | 1.21 |
|  | 0.66 |
|  | 1.04 |
|  | 0.69 |
| L3 Larvae | 223.50 |
|  | 227.33 |
|  | 308.32 |
|  | 272.09 |
|  | 270.38 |
| L3/L4 Larvae | 128.91 |
|  | 200.62 |
|  | 163.25 |
|  | 179.13 |
|  | 100.76 |
| L4 Larvae | 80.89 |
|  | 76.99 |
|  | 91.33 |
|  | 81.72 |
|  | 88.94 |
| Male pupae | 10.91 |
|  | 4.20 |
|  | 2.30 |
|  | 8.52 |
|  | 1.57 |
| Female pupae | 12.86 |
|  | 6.85 |
|  | 2.23 |
|  | 1.62 |
|  | 1.78 |

| Rearing water sample | Residue in Sample (µg/mL) |
| --- | --- |
| Tap water control | 0.09 |
| LOQ spike | 0.12 |
| 10x LOQ spike | 0.54 |
| 100 x LOQ spike | 4.74 |
| L1/L2 rearing water | 0.11 |
|  | 0.10 |
|  | 0.09 |
|  | 0.09 |
|  | 0.09 |
| L3 rearing water | 8.09 |
|  | 9.09 |
|  | 9.18 |
|  | 7.73 |
|  | 7.89 |
| L3/L4 rearing water | 2.79 |
|  | 2.65 |
|  | 0.10 |
|  | 2.55 |
|  | 2.49 |
| L4 rearing water | 2.41 |
|  | 1.83 |
|  | 1.43 |
|  | 1.71 |
|  | 2.39 |
| Pupae rearing water | 1.23 |
|  | 1.13 |
|  | 2.19 |
|  | 1.59 |
|  | 1.02 |
