# Supplementary material for: Assessment of the Impact of Potential Tetracycline Exposure on the Phenotype of Aedes aegypti OX513A: Implications for Field Use
Source: PLoS Negl Trop Dis. 2015 Aug 13;9(8):e0003999. doi: 10.1371/journal.pntd.0003999 (PMC4535858; doi:10.1371/journal.pntd.0003999)
Supplement: S3 Table — (DOCX) [file pntd.0003999.s004.docx]

**S3 Table. GPS coordinates of water sampling points in Brazil.**

| Sampling point | South Coordinate | West Coordinate |
| --- | --- | --- |
| Pinheiros Creek | -22,9125 | -46,9597 |
| Anhumas Creek | -22,7698 | -47,0953 |
| Piçarrão Creek | -22,9360 | -47,1626 |
| Poultry CAFO | -23,1409 | -46,6046 |
| Fish production lake | -22,7841 | -47,0832 |
| Larval habitat 1 | -22.9977 | -47.0894 |
| Larval habitat 2 | -22.9974 | -47.0866 |
| Larval habitat 3 | -22.9984 | -47.0863 |
| Larval habitat 4 | -22.9978 | -22.0874 |
| Larval habitat 5 | -22.9975 | -47.0866 |
| Larval habitat 6 | -22.9983 | -47.0864 |
| Campinas (rain and tap water) | -22,7949 | -47,0762 |
| Itu (rain and tap water) | -23,2746 | -47,2897 |
