# Supplementary material for: Assessment of the Impact of Potential Tetracycline Exposure on the Phenotype of Aedes aegypti OX513A: Implications for Field Use
Source: PLoS Negl Trop Dis. 2015 Aug 13;9(8):e0003999. doi: 10.1371/journal.pntd.0003999 (PMC4535858; doi:10.1371/journal.pntd.0003999)
Supplement: S4 Table — oxytetracycline (OTC), tetracycline (TC) and chlortetracycline (CTC). (DOCX) [file pntd.0003999.s005.docx]

**S4 Table. Retention time, MRM transitions, fragmentor voltage and collision energy (CE) for each tetracycline analyte;** oxytetracycline (OTC), tetracycline (TC) and chlortetracycline (CTC).

| Compound | t_R_ (min) | Fragmentor (V) | MRM transition | CE (V) |
| --- | --- | --- | --- | --- |
| OTC | 6,58 | 110 | 461.1 to 427.2  461.1 to 444.2  461.1 to 201.0 | 0  10  20 |
| TC | 6,49 | 120 | 445.1 to 154.0  445.1 to 411.1  445.1 to 428.1 | 20  10  0 |
| CTC | 7,48 | 120 | 479.1 to 154.1  479.1 to 445.0  479.1 to 463.0 | 10  10  20 |
