# Supplementary material for: Assessment of the Impact of Potential Tetracycline Exposure on the Phenotype of Aedes aegypti OX513A: Implications for Field Use
Source: PLoS Negl Trop Dis. 2015 Aug 13;9(8):e0003999. doi: 10.1371/journal.pntd.0003999 (PMC4535858; doi:10.1371/journal.pntd.0003999)
Supplement: S5 Table — Raw data. Refer to Table 1 for information on treatment. (DOCX) [file pntd.0003999.s006.docx]

**S5. Table. Effects of adult ingestion of chlortetracycline on OX513A progeny.** Raw data. Refer to Table 1 for information on treatment.

| Treatment | Dead pupae | Dead adults on water | Dead adults on cage | Non flying adults | Flying adults |
| --- | --- | --- | --- | --- | --- |
|  |  |  |  |  |  |
|  |  |  |  |  |  |
| A | 79 | 40 | 10 | 3 | 4 |
|  | 90 | 33 | 10 | 3 | 4 |
|  | 118 | 31 | 6 | 1 | 5 |
|  | 106 | 14 | 5 | 2 | 5 |
|  | 97 | 25 | 1 | 1 | 3 |
|  | 121 | 25 | 6 | 0 | 2 |
| B | 79 | 45 | 9 | 2 | 1 |
|  | 79 | 41 | 5 | 2 | 4 |
|  | 124 | 29 | 7 | 0 | 5 |
|  | 96 | 32 | 1 | 0 | 5 |
|  | 90 | 25 | 12 | 0 | 4 |
|  | 69 | 26 | 2 | 0 | 6 |
| C | 102 | 39 | 3 | 0 | 3 |
|  | 96 | 34 | 10 | 2 | 7 |
|  | 117 | 29 | 3 | 0 | 1 |
|  | 125 | 28 | 6 | 2 | 4 |
|  | 107 | 32 | 6 | 0 | 3 |
|  | 101 | 35 | 0 | 0 | 4 |
| D | 101 | 38 | 8 | 1 | 5 |
|  | 101 | 30 | 14 | 0 | 5 |
|  | 91 | 30 | 6 | 3 | 5 |
|  | 100 | 26 | 18 | 2 | 2 |
|  | 99 | 39 | 9 | 3 | 2 |
|  | 94 | 32 | 9 | 0 | 2 |
| E | 111 | 39 | 6 | 0 | 7 |
|  | 109 | 30 | 7 | 1 | 9 |
|  | 112 | 21 | 23 | 2 | 6 |
|  | 126 | 22 | 12 | 1 | 4 |
|  | 103 | 16 | 21 | 2 | 14 |
|  | 101 | 26 | 17 | 0 | 12 |
| F | 1 | 0 | 2 | 0 | 89 |
|  | 0 | 0 | 0 | 0 | 83 |
|  | 1 | 4 | 3 | 0 | 160 |
|  | 0 | 32 | 1 | 3 | 156 |
|  | 1 | 1 | 0 | 0 | 80 |
|  | 0 | 5 | 2 | 0 | 78 |
| G | 1 | 14 | 5 | 0 | 127 |
|  | 0 | 23 | 2 | 0 | 151 |
|  | 1 | 3 | 0 | 0 | 181 |
|  | 0 | 4 | 6 | 2 | 144 |
|  | 0 | 2 | 0 | 0 | 176 |
|  | 0 | 8 | 0 | 0 | 112 |
